# Building Climate Resilience in the Blue Nile/Abay Highlands: A Role for Earth System Sciences

**DOI:** 10.3390/ijerph9020435

**Published:** 2012-01-30

**Authors:** Benjamin F. Zaitchik, Belay Simane, Shahid Habib, Martha C. Anderson, Mutlu Ozdogan, Jeremy D. Foltz

**Affiliations:** 1 Department of Earth and Planetary Sciences, Johns Hopkins University, Baltimore, MD 21210, USA; 2 College of Development Studies, Addis Ababa University, Addis Ababa, Ethiopia; Email: simaneb@yahoo.com; 3 Office of Applied Sciences, NASA Goddard Space Flight Center, Greenbelt, MD 20770, USA; Email: shahid.habib@nasa.gov; 4 Hydrology and Remote Sensing Lab, USDA Agricultural Research Service, Beltsville, MD 20705, USA; Email: martha.anderson@ars.usda.gov; 5 Forest and Wildlife Ecology, University of Wisconsin-Madison, Madison, WI 53706, USA; Email: ozdogon@wisc.edu; 6 Agricultural and Applied Economics, University of Wisconsin-Madison, Madison, WI 53706, USA; Email: jdfoltz@wisc.edu

**Keywords:** climate change, adaptation, resilience, land degradation, erosion

## Abstract

The Blue Nile (Abay) Highlands of Ethiopia are characterized by significant interannual climate variability, complex topography and associated local climate contrasts, erosive rains and erodible soils, and intense land pressure due to an increasing population and an economy that is almost entirely dependent on smallholder, low-input agriculture. As a result, these highland zones are highly vulnerable to negative impacts of climate variability. As patterns of variability and precipitation intensity alter under anthropogenic climate change, there is concern that this vulnerability will increase, threatening economic development and food security in the region. In order to overcome these challenges and to enhance sustainable development in the context of climate change, it is necessary to establish climate resilient development strategies that are informed by best-available Earth System Science (ESS) information. This requirement is complicated by the fact that climate projections for the Abay Highlands contain significant and perhaps irreducible uncertainties. A critical challenge for ESS, then, is to generate and to communicate meaningful information for climate resilient development in the context of a highly uncertain climate forecast. Here we report on a framework for applying ESS to climate resilient development in the Abay Highlands, with a focus on the challenge of reducing land degradation.

## 1. Introduction

Coupled processes of low investment capacity and land degradation currently drive a cycle of depressed agricultural yields and persistent poverty through much of the Ethiopian Highlands, including the Blue Nile (Abay River) Highlands (BNH) region. There is reason for concern that conditions in the BNH will deteriorate in coming decades, given a changing climate that could well bring more frequent drought and more intense precipitation events to the region [1]. This is expected to take place in tandem with continued population growth and the potential for external land use pressures associated with hydropower development and commercial agriculture. These external stresses are being imposed on a local economy that is almost entirely dependent on agriculture and that has exhibited significant swings in crop productivity and economic output in response to recent interannual climate variability [2,3,4,5]. 

**Figure 1:**
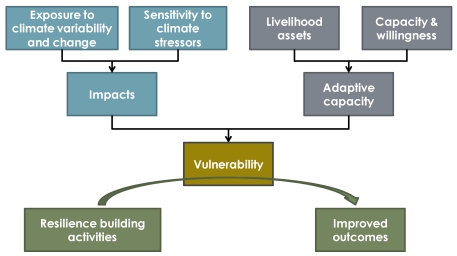
Conceptual framework for climate resilience in the BNH.

As described in detail in Simane *et al.* [6], the recognized links between climate variability, food and economic security, and development capacity in the BNH have motivated a significant effort of research and applications aimed at building greater climate resilience in the area. In this context, we examine climate *resilience* at the community level, defining it as the ability of communities to withstand and recover from climate-induced stresses. Communities can engage in resilience-building activities as a response to identified *vulnerability* to climate change, which is in turn a function of climate *impacts* mediated by the community’s *adaptive capacity* (Figure 1). One can think of resilience-building activities as a means to enhance development in a hostile climate [7], including actions designed specifically to reduce climate impacts as well as investments in basic infrastructure, services, and education that serve to increase adaptive capacity. Figure 1 delineates the linkages between climate impacts, adaptive capacity, and resilience building activities and how they can mediate vulnerability to climate events.

Within this conceptual framework, Earth System Science (ESS) has the potential to contribute to the characterization and projection of climate exposure and sensitivities in the region and to inform the design of effective resilience-building strategies that are consistent with physically-based understanding of present and future climate impacts. These potential contributions are, however, constrained by a paucity of long-term environmental monitoring records in the BNH, which limit researchers’ ability to diagnose trends and to evaluate model performance, as well as by significant uncertainties in model projections of future climate for the region. While these constraints are the subject of major research and capacity building effort, they are likely to remain for quite some time: building long-term data records requires not only investment but also *time*, and the production of more accurate climate projections is limited by inherent uncertainties in emissions trajectories as well as by considerable and possibly irreducible uncertainties in the simulation of climate feedbacks on the local to global scale. As a result, climate adaptation will have to take place in the context of uncertain climate forecasts [8]. Making the most out of the information that is available, including information on uncertainty, is a major challenge for all stakeholders concerned with building climate resilience.

Building on the outcomes of a recent expert workshop on *Climate Resilience in the Blue Nile Highlands* (Bahir Dar, Ethiopia, 8–11 July 2011), this paper presents a model for confronting the challenge of developing ESS-informed climate resilience strategies for the BNH in the face of limited data, complex coupled human-environmental systems, and significant uncertainty in climate projections. As the potential for accelerating land degradation has been identified as a major climate adaptation priority for the region, both at the expert workshop and in Ethiopia’s National Adaptation Plan for Action [9], we consider the problem of water-driven soil erosion in the BNH as a case study of ESS capabilities and needs with respect to climate resilient development.

Section 2 of the paper reviews approaches to ESS-based climate resilience analysis in the context of uncertainty. Section 3 describes the geography, climate, and prevailing economic conditions of the BNH. Section 4 presents a framework for developing and applying ESS information in support of improved land management under climate change. Conclusions are offered in Section 5.

## 2. Building Resilience under Uncertainty

### 2.1. The Challenge of Uncertainty

**Figure 2:**
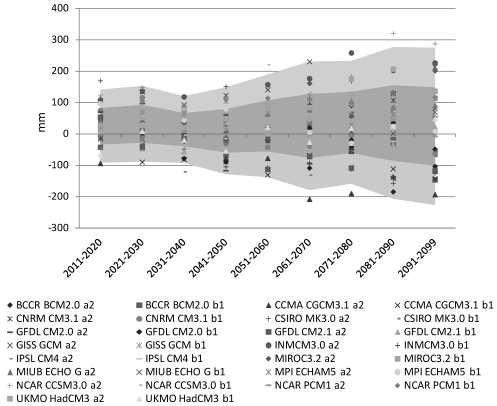
Change in average annual precipitation (mm) for decades of the 21st century, averaged over the BNH, relative to the 1961–1990 mean. Model data from the CMIP3 archive [12], processed using climate wizard [19]. Shading indicates one and two standard deviations from the multi-model ensemble mean.

The development of advanced Global Climate Models (GCMs) has yielded enormous insight regarding the mechanisms governing climate variability and anthropogenic climate change. The models have also provided perspective on some of the critical anticipated impacts of 21st century climate change on the global scale: significant increases in temperature across most of the planet, amplified warming and accelerated melt in cold lands, and an amplification of zonal precipitation contrasts—including substantial drying in the subtropics—are all robust results that appear consistently across models and, increasingly, in the observational record [10,11]. At the same time, the results of multi-model GCM comparison studies such as the Coupled Model Intercomparison Projects—notably CMIP3 [12], which served as the basis for GCM results assessed in the Intergovernmental Panel on Climate Change (IPCC) 4th Assessment Report (AR4) and emerging results from CMIP5 [13], which will provide critical model results for the 5th Assessment Report (AR5)—have exhibited large uncertainty in regional to subregional precipitation [14]. This uncertainty is clearly evident in the BNH, which falls in a region of particularly high uncertainty in projections of precipitation change (Figure 2). While it may be possible to weight these model projections on the basis of current model performance, most studies have found that credibility rankings of GCMs do not contribute significantly to reduced uncertainty in climate projections [15,16,17,18]. Moreover, uncertainty in GCM predictions is only the first component in a cascade of uncertainties encountered when attempting to predict climate impacts relevant to society. For example, the agricultural impacts of climate change in a mountainous area will depend on the influence of global change on localized climate conditions and on how the physical, ecological, agricultural, and economic systems might respond to those changes both with and without adaptive action. 

There are extensive efforts underway to improve the skill of climate models, raising the possibility that the spread in future predictions will ultimately be reduced. It is unlikely, however, that the range of uncertainty in regional predictions will be narrowed to any significant extent in the near future. To some degree this is a product of irreducible uncertainties in emissions scenarios [20,21], which have a major impact on climate projections in the second half of the 21st century. It is also a product of divergent representation of thresholds and feedbacks between GCMs that are equally credible at the global scale, and of the computational difficulty of running large ensembles of GCMs at a spatial resolution capable of resolving local atmospheric processes. Additionally, as GCMs increase in sophistication, there is a tendency for model divergence to *increase* as additional coupled processes are included in increasingly complex modeling systems. There is an active debate in the field of climate modeling as to the most appropriate modeling and analytical strategies for addressing the predictability challenge [22,23,24], but the implication for applied climate adaptation communities is clear—decision making in the context of considerable uncertainty in global forecast products will be a reality for years to come, and perhaps indefinitely. 

It should be noted that some adaptation can take place in the absence of skillful climate projections, in the form of so-called “no regret” investments that make a contribution to development independent of the climate change signal (e.g., child education). In the “no regrets” paradigm, ESS is essential in identifying climate change as a potential threat, thus adding urgency to the development challenge, but ESS information derived from climate projections is not required for adaptation: the proposed investments will yield a positive return under any climate scenario.

While the term “no regret” investments is not a perfect descriptor—any investment comes at a cost, and therefore with the potential for regret should higher priorities arise—it is certainly true that investments that yield a development benefit independent of the climate signal deserve high priority in any development strategy. Additionally, many “no regret” investments do address issues of climate variability and hydrometeorological disasters that are directly relevant to climate change preparedness. But there are abundant examples of the limitations of relying on no regrets approaches to climate resilience. These limitations apply to centralized decisions such as large infrastructure developments (e.g., hydroelectric dams), transboundary water agreements, biodiversity preservation strategies, and agricultural research programs. They also affect diffuse adaptation challenges, including on-farm cropping decisions, evolving disease risk and water security considerations, and risk management across space and time under changing patterns of extreme weather events. In all of these areas, ESS information is critical for climate resilient development.

### 2.2. Making Use of an Uncertain Forecast

One way in which uncertain climate projections can be applied to enhance climate resilience is to recognize that planning for climate change is fundamentally a process of risk characterization rather than a problem for deterministic prediction. In this respect, applying climate model information to adaptation is not unlike many other scenario-based planning processes. Climate projections from GCMs provide scenarios that inform analysis of potential outcomes, allowing stakeholders to evaluate their risk exposures and adaptation options. Such a risk-based approach differs from conventional vulnerability assessments in that it includes a formal assessment of likelihood of impacts, clearly defined for a sector, time horizon, and time scale (*i.e.*, events, variability, and/or trends) of interest [25].

From a technical ESS perspective, a recent study on drought in the United States by Strzepek *et al.* [26] provides an informative example of research results applied in a risk characterization framework. The authors of the study note that previous analyses of climate change impacts on U.S. drought, though useful from a scientific perspective, had not simultaneously considered the range of drought definitions and the range of GCM uncertainty in the evaluation of future drought risk. Furthermore, earlier studies employed a range of downscaling methods to process GCM results. While downscaling may be necessary for some applications, it adds an additional layer of uncertainty and the potential of poorly constrained error analysis that complicates objective risk analysis. By clearly defining the impacts of interest and the scale of analysis, Strzepek *et al.* were able to construct an ESS study that focused on clearly defined metrics, that targeted selected time periods and climate scenarios of interest, and that approached GCM data in a consistent manner on an appropriate spatial scale. The result was a national scale drought risk assessment that identified regions at greatest risk according to various drought metrics. Interestingly, it was found that the frequency of meteorological drought is expected to increase in some regions of the U.S., while decreasing in others, but that the frequency of hydrological drought is expected to increase across almost all of the country. From a risk management perspective, then, the study provides a foundation for targeting further studies to high risk regions and also helps decision makers to distinguish between highly uncertain, spatially variable meteorological drought trends and more consistent predictions regarding hydrological drought. 

The same general methodological approach: identify a potential impact, define the metrics of impact and scale of analysis, run climate scenarios, and characterize risk and uncertainty, can be applied to make adaptation recommendations in a developing country setting such as the BNH. The “total risk assessment” studies performed by the Economics of Climate Adaptation Working Group (ECA 2009), for example, employed the approach to examine ESS-informed climate decision making across a range of development and climatic environments. For a case study in central Mali, they focused on potential climate impacts on agricultural returns, quantified in terms of changes in local and national agricultural income. The risk of declining returns was assessed using climate-driven crop and livestock models, with scenarios run through 2030. Like the BNH, Mali lies in a region of large uncertainty in predicted precipitation change, with models predicting anything between a 10% increase and a 10% decrease in annual rainfall by 2030. From the perspective of agricultural income, however, both high precipitation and low precipitation scenarios were found to represent a significant risk: the dry scenario would be expected to cause a 15% drop in national agriculture and livestock income relative to a scenario of no climate change, while the “best case” scenario—a shift towards higher rainfall—was still associated with a 5% drop in predicted income. 

The ESS-based risk assessment for Mali indicates, therefore, that investments in technological change for local agriculture will be necessary in order to avoid climate-related economic losses in the absence of large scale migration of populations to regions of lower climate sensitivity. Even accounting for the uncertainty in rainfall, a broad range of cost positive climate-informed investments can be proposed that would be expected to enhance resilience, including shifts to crops that are robust to predicted climate scenarios and improved water management infrastructure that will be required even in the presence of a net rainfall increase, on account of the predicted timing and severity of rains under the high precipitation scenario. With the benefit of ESS-informed risk analysis, which made use of climate projections, crop and livestock models, and integrated economic analysis, these adaptation options can be evaluated against and prioritized with respect to other development investments available at local and national scale.

**Figure 3:**
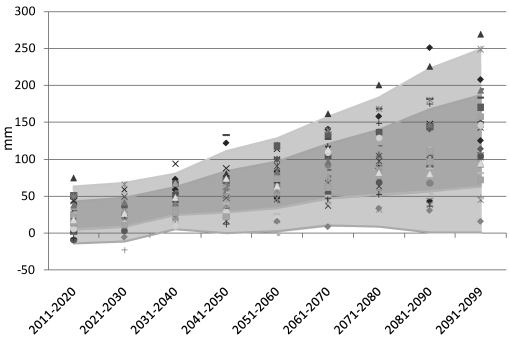
As in Figure 2, but for the projected change in annual soil moisture deficit (mm). The ensemble result provides high confidence that soil moisture deficit will increase in the BNH.

These experiences in risk characterization raise a second important point about the use of climate projections to inform decision making: GCM predictions are not divergent in all variables. It is important, therefore, to define clearly the aspects of climate that matter most to an outcome of interest at the outset of any risk analysis [27]. In the case of U.S. drought analysis [26], a GCM ensemble that was highly uncertain with respect to short-term meteorological drought (a function of precipitation only) was convergent in the projection of longer-term hydrological drought (a function of precipitation, potential evapotranspiration, and soil characteristics). In the ECA study of Mali, projections that diverged on precipitation yielded the same directional result with respect to the impact of interest: agriculture income was projected to decline. Similar distinctions in predictability apply to climate change impacts in the BNH. For example, while annual precipitation predictions for the BNH show a wide spread (Figure 2), model agreement is stronger for critical variables such as precipitation intensity, potential evapotranspiration, and soil moisture deficit, all of which can be applied to assess specific risks related to water resources, agriculture, human health, and other issues that are the true target of adaptation efforts. Soil moisture deficit (Figure 3), for example, is associated with the fraction of potential evapotranspiration that is recognized in actual evapotranspiration. This variable is regularly applied to irrigation scheduling and in yield forecasts for rainfed crops.

## 3. The Blue Nile Highlands

### 3.1. Local Conditions

#### 3.1.1. Climate

**Figure 4:**
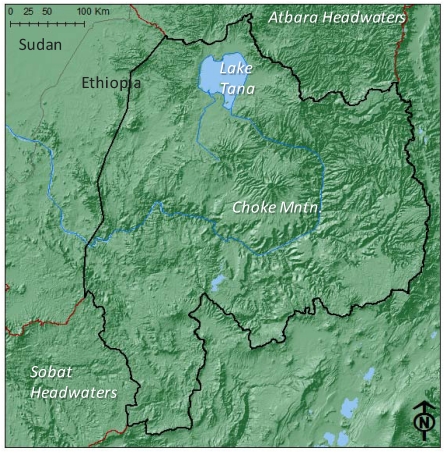
The BNH, outlined in black, are defined here to include the entire Blue Nile watershed above 800 m elevation.

**Figure 5:**
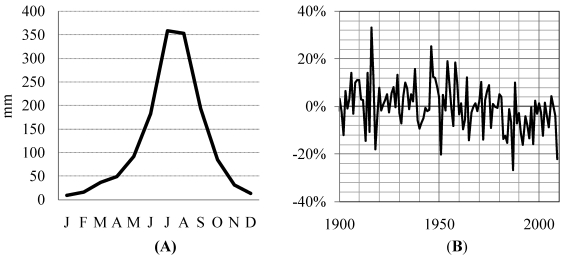
(**A**) Climatological monthly precipitation and (**B**) annual percent anomaly in rainy season (June–September) precipitation in the BNH, extracted from FEWSNET precipitation analysis [1,34].

The BNH are located in the area surrounding 10°N, 36°E, and have elevation ranging from approximately 800–4200 meters above sea level (Figure 4). The prevailing climate is tropical highland monsoon [28]. Seasonal precipitation is tightly correlated with the movement of the Inter-tropical Convergence Zone (ITCZ), with most rain falling during the May–October *kiremt* rainy season (Figure 5A). The distribution of precipitation within the BNH is far from uniform; average annual precipitation ranges from 600 to 2000 mm·yr^−1^ and exhibits strong local variability associated with topographic gradients [29]. Precipitation events are convective in nature, characterized by short, sometimes intense erosive bursts with notably large raindrops [30]. Interannual variability in precipitation is significant (Figure 5B), and has major impacts on agricultural production [3] and soil erosion rates. Variability on this timescale is strongly associated with the El Nino Southern Oscillation (ENSO) and, perhaps, with Indian Ocean SST [31,32]. The Atlantic and Pacific Decadal Oscillations also impact atmospheric dynamics in East Africa, and they appear to have some influence on BNH precipitation [33]. 

As noted above, GCMs yield a wide range of predictions for future precipitation patterns in the BNH. Beyene *et al.* [35] analyzed GCM simulations included in the IPCC 4th Assessment Report and found that most models predict a transient increase in precipitation in the first half of the 21st century, followed by a decline after 2050. But this is far from a robust prediction, as evident in Figure 2. 

#### 3.1.2. Soils and Hydrology

Soils of the western BNH include reddish Nitosols and Acrisols on deeply weathered bedrock, while Phaeozems and Andosols dominate at higher altitudes and in the northern portion of the region. Vertisols tend to form at footslopes throughout the region [36]. Under undisturbed conditions, soils tend to be deep: natural depths are thought to be more than 50 cm over most of the BNH, with rooting depths up to one meter [37], allowing for local variability across terrain. These deep, weathered tropical soils are highly susceptible to erosion, and on lands cultivated using traditional methods the rate of soil loss can exceed the rate of soil generation by a factor of 4 to 10 [37], due in large part to the prevalence of traditional ox-drawn tillage systems that promote rapid erosion [36]. High rates of on-field erosion are particularly problematic given that nutrients in BNH soils tend to be concentrated in the upper portion of the soil column. On-field soil loss also leads to reduced water holding capacity and faster concentration of water on the landscape, which contributes to large volume gully erosion and sediment transport [38]. Combined erosion processes across the BNH have led to dramatic soil erosion, particularly from steep slopes, and significant loss of soil productivity. The FAO has estimated an average rate of soil loss of 100 tons·ha^−1^·yr^−1^ (or 8 mm depth) averaged across the Ethiopian Highlands [39], with significantly higher rates of erosion identified in the northwest region that includes portions of the BNH [40]. This physical transformation has serious implications for food security. For example, Tekatay [40] estimates that in 1990 alone, reduced soil depth due to erosion resulted in production loss of 57,000–128,000 tons in Ethiopia—a volume that would have been sufficient to feed more than four million people. Nevertheless, there are still significant soil resources in the BNH, and experience indicates that productivity can be maintained and enhanced through effective field scale and landscape scale land management [41,42].

#### 3.1.3. Agricultural Systems

Agriculture in the BNH is predominantly crop-livestock mixed systems, practiced by independent farmers on small plots. As an example, farms in agricultural systems in 16 watersheds around Choke Mountain (location shown in Figure 4) average 0.5 hectares in size, with cultivation including tef, maize, and wheat for market and additional crops such as barley and potato produced for home or local consumption [43]. Animals include cows, oxen, sheep, and horses. A defining characteristic of cropping systems in the BNH, and throughout the Ethiopian Highlands, is the use of the ancient Ethiopian *ard* (or *maresha*) plow for tillage. This simple wooden ox-drawn plow is well suited for tropical vertic soils because it breaks through hard, dry topsoils [36]. It is also, however, an instrument associated with tillage practices that lead to high rates of on-field erosion, particularly on steep slopes. Numerous studies have noted that the stagnation of agricultural investment and innovation, exemplified by widespread use of the Ethiopian *ard*, is a strong explanatory factor for high erosion rates in the BNH, and that improved farming techniques, including more modern tillage practices, are capable of slowing, and even reversing, the cycle of erosion, low yields, deforestation, and further land degradation [36,44,45]. A concerted effort to improve tillage practices, to match cropping zones with optimal environmental niches, and to expand nascent sustainable agricultural techniques, including agroforestry, has been proposed as a remedy to poor agricultural conditions in the BNH, though further analysis is required to understand the potential of these efforts to contribute to physically and economically sustainable landscapes. 

#### 3.1.4. Socio-Economic Conditions

As in most of rural Ethiopia, the economy of the BNH region is highly dependent on agriculture. Smallholder farmers form the backbone of the Ethiopian agricultural sector, cultivating 95% of the cropped area, and producing 90–95% of the country’s cereals, pulses and oilseeds. Smallholder farmers are principally concerned with meeting their subsistence needs. In the most productive areas any surplus produce will be sold, but the amounts are usually limited, while in the more marginal areas many farm households struggle to meet their own annual food needs. The performance of the crop production sub-sector over the last 30 years has been poor. It has failed to keep up with the demand from a growing population, as per capita food grain production has steadily decreased over this period. Whereas in the past Ethiopia had been self-sufficient in food, and a net exporter of food grains, it has been a net importer of grain since 1981/82. Population pressure, particularly in highland farming areas like the BNH, has led to a decline in farm size. This trend, combined with increasing land degradation and recurrent droughts, has contributed to declining crop productivity. The problem has been exacerbated by such factors as insecure land tenure, weak agricultural research and extension services, and inadequate input supply and produce marketing systems.

Land tenure in Ethiopia is, in general, perceived to be somewhat insecure, due to a historical government policy of periodic land redistributions to allocate land to land-poor households; this policy was only recently abandoned by the government. The impacts of insecurity or perceived insecurity on agriculture investments differ for different types of agricultural investment [46]. Deininger *et al.* suggest that land redistributions led to a transient increase in quick-reward agricultural inputs, as land was redistributed to households that had the means and desire to invest in fertilizer and equipment, but that long-term insecurity caused by redistribution policies resulted in less frequent fallowing, lower investment in conservation measures, and increased soil degradation [47]. Other studies, however, have suggested that access to resources might be more important than land tenure concerns in some cases [48]. Researchers have found that farmer investments are affected by land tenure concerns even in the absence of land redistributions, since increasing population leads to the expectation of reduced farm size as land are divided up between family members. Land transfer rights may also influence investment patterns and cropping choices. Interestingly, perceptions of tenure security and land transfer rights are often at odds with the actual legal status of lands [49]. In this respect, perceptions of land tenure may be a predictor of farmer investment in adaptation strategies that require investments in the land. 

### 3.2. Regional Context

The condition of the BNH is of critical interest to all three nations that share the Blue Nile River—Ethiopia, Sudan, and Egypt. In total, 86% of average Nile River flow at Aswan (Egypt) originates in the Ethiopian Highlands: about 64% from the Blue Nile and the remainder from the Atbara and Sobat Rivers. This fact is frequently cited in the context of regional tension over the Nile, as downstream riparian nations have sometimes expressed suspicion of and outright opposition to Ethiopian plans to develop the water resources of the BNH. In recent years this tension has risen to the highest political levels in the negotiations of the Nile Basin Initiative (NBI), a 10-nation consortium established to promote joint management of the combined Nile basin [50]. 

In this context, improved water management in the BNH takes on delicate regional political dimensions. Sustainable practices in the BNH can, however, provide a benefit to all people who share the Nile. Erosion in the BNH results in a tremendous sediment load in the river, with approximately 140 million tons of sediment reaching Sudan every year [51]. In historic times, sediment delivered with the annual flood provided fresh soil to agricultural systems in Sudan and Egypt. This system ended with the construction of large hydropower dams on the Nile, including the High Aswan Dam in Egypt and the Roseires Dam in Sudan. Under current conditions, sediment delivered in the Blue Nile is a significant burden to downstream users of the river: Sudan spends millions of dollars every year dredging sediment out of reservoirs and irrigation canals, and is currently in the process of raising the height of the Roseires Dam to make up for the fact that sediment has reduced that reservoir’s holding capacity from 3.3 to less than 1.9 BCM in the 45 years since the dam was constructed. Reduced water holding capacity in the BNH also leads to flashy flow in the Blue Nile, with reduced baseflow during the dry season and the potential for more catastrophic floods during the *kiremt*. This loss of natural water holding capacity increases the challenge of reservoir storage downstream. With construction of the multi-billion dollar Ethiopian “Grand Renaissance Dam” now underway on the main stem Blue Nile near the border with Sudan, erosion control in the BNH has become an even more significant concern for Ethiopia as well.

The BNH, and the Ethiopian Highlands more broadly, also have an important role to play in regional food security. Even considering the extensive degradation of highland slopes that has occurred over time, the BNH still has the advantages of naturally deep and fertile soils and ample precipitation. In East Africa, where lowland drought can cause devastating crop failure and famine on a regular basis, and where downstream Nile riparians rely on massive irrigation schemes and, increasingly, food imports to guarantee food security, enhanced production in the BNH represents an important opportunity. While steep hillsides within the BNH are not appropriate for large-scale industrial agriculture of the type usually associated with breadbasket regions, there are large fertile extents within the BNH that are entirely capable of producing cereals and other crops that would sustain the local population with excess to be placed on markets around the region. 

Food security is, of course, intimately related to the availability of productive agricultural lands. Population in Ethiopia, and throughout East Africa, continues to grow at one of the fastest rates in the world. At the same time, land degradation threatens fertility in many traditional production zones, including the BNH, and external land pressures in the form of large long-term land leases to investors from Asia and the Middle East represent an emerging constraint on agricultural development options at the national level (though such leases are not an issue within the BNH). It should be noted that perspectives on these land leases differ; while the long-term lease of land and water resources to external, export-minded investors raises concerns in a food insecure region, these investors also bring capital and capacity to the region, promoting higher production on underutilized land.

With respect to water resources, food security, and land availability, then, the BNH are in communication with dynamics in the broader region. In each case, the implications of this communication for climate resilient development in the BNH are uncertain. Improved understanding of the vulnerabilities of the BNH to these stresses, in the context of a changing climate, and identification of opportunities for enhanced resilience, are a critical need.

## 4. Climate Change and Land Degradation

**Figure 6:**
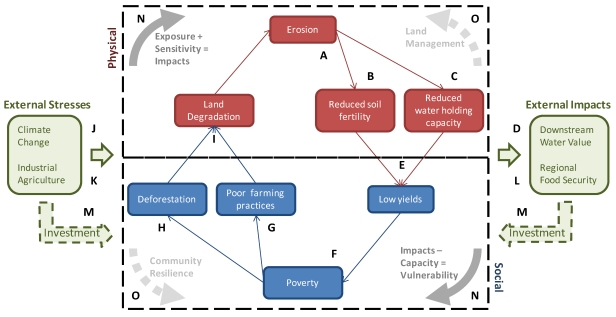
Framework for reversing coupled degradation-vulnerability processes in the context of climate change.

The conditions in the BNH described above—landscapes characterized by steep, dissected terrain and highly erodible soils, with fragmented land use dominated by small-holder crops and livestock production intermixed with shrinking residual forest stands—are associated with a coupled cycle of land degradation and poverty (Figure 6). Due to naturally erosive rains and erodible soils, the area is highly susceptible to erosion (A), which leads to reduced water holding capacity and lost soil fertility locally (B,C) and to high sediment loads and flashy flow in the Blue Nile, which reduces water quality and necessitates intensive canal and reservoir management downstream (D). Degraded soils contribute to low agricultural yields (E), and, as agriculture is the economic base of the region, to persistent poverty (F). Poverty, combined with related land tenure challenges, leads to low investment capacity, with the result that traditional farming practices are employed throughout the region (G), and residual forest stands are lost as the expanding poor population brings more land into agriculture (H). Poor practices at the field scale and deforestation at the landscape scale promotes further land degradation (I), worsening erosion.

This local coupled system is also affected by external stresses. Climate variability and, increasingly, climate change (J) result in more variable and less predictable weather patterns, including more erosive rains. Climate change is also expected to affect the distribution of agro-ecological zones, influencing the yield potential of crops across local environments. External land use dynamics (K) are also relevant, as large-scale land leases and other industrial-scale agricultural development exert a forcing on commodity prices as well as on the value assigned to land and water in the BNH. Finally, yields in the BNH, one of the primary “water towers of Africa,” are intimately related to national and regional food security (L), which, alongside water management in the Nile Basin, external land use dynamics, and interest in effective climate change adaptation, offers opportunities for investment in the BNH (M). As the impacts of climate change threaten to increase vulnerability in low capacity communities (N), this external investment, together with local capacity building efforts, offer an opportunity to reverse the cycle of land degradation and enhance climate resilient development (O). These opportunities are, however, constrained by the magnitude and the rate of climate change, as well as by existing biophysical and, in some cases, socio-cultural limitations on adaptive potential.

The primary interactions of climate variability and change with this coupled system, then, include the influence of climate on agricultural production and returns, on erosion potential, on downstream water value, on food production and demand patterns at the regional scale, and on international development investments and aid. Note that within his framework the direct impact of climate on erosion potential is one point of interaction with land degradation, but it is not the only point of interaction and may, indeed, be a secondary concern relative to the impacts that climate will have on agro-economic conditions that drive land management patterns. Focusing on ESS aspects of the interaction points local to the BNH (agricultural production, erosion potential, and downstream water value), we find that current climate analysis tools can provide substantial information for climate resilient development strategies, even considering the wide spread of future precipitation projections. Where information is incomplete, it is possible to characterize risk and uncertainties in a meaningful way and to direct future research towards the most relevant gaps in knowledge.

### 4.1. Agricultural Production

Land cover and land use practices are the primary drivers of human-induced soil erosion in agricultural landscapes across the world. As such, an evaluation of climate change impacts on land degradation must include some assessment of how climate change will affect agricultural production for a range of crops and farming activities. Projections of on-farm productivity provide estimates of changing land value for production under various cultivation options. They also contribute to multidisciplinary evaluations of potential land use trends in coming decades, and they can be used to inform research and investment strategies targeted to address shifting agricultural opportunities and vulnerabilities. 

A number of studies have investigated the impact of historical climate variability on crop yields in Ethiopia [3,4,5], with some attention to specific impacts on the Highlands zone. These studies have consistently demonstrated that Ethiopian agricultural production is highly sensitive to interannual variability in precipitation, in large part because agriculture is almost entirely rainfed across the country. As agriculture accounts for over 40% of Ethiopian GDP, this sensitivity has a significant impact on the national economy [2], with even larger localized impacts in zones affected by precipitation extremes.

**Figure 7:**
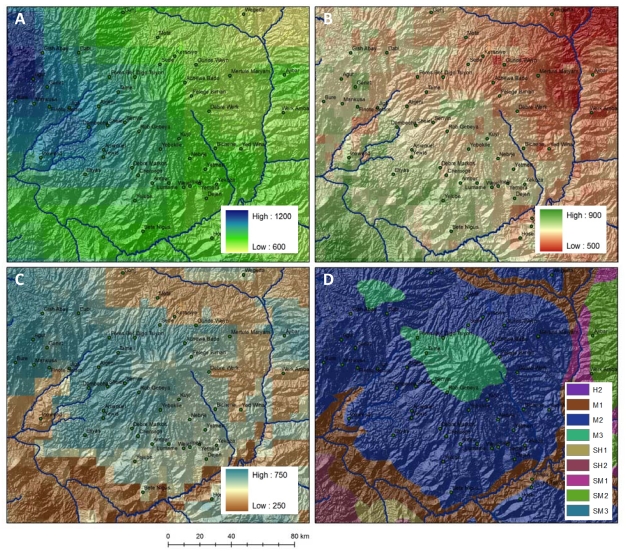
(**A**) Topographically-downscaled satellite derived precipitation estimates (mm), (**B**) simulated evapotranspiration (mm), (**C**) integrated soil moisture content to 2 meters depth (mm), and (**D**) defined agroecological zones for Choke Mountain. (**A**–**C**) are 10 yr climatological annual averages generated with the NASA Nile Land Data Assimilation System [54]. In (**D**), H = humid, M = moist, SH = sub-humid, SM = sub-moist.

These studies provide a valuable platform for analyzing climate impacts on agricultural production in the BNH, but participants in the *Climate Resilience in the Blue Nile Highlands* workshop determined that significantly more ESS research was required. Workshop participants considered current levels of information provided by crop models inadequate in both accuracy and coverage. This suggests that future research should work to establish a more informative baseline based on historical data. Models of crop performance—either simplified crop yield factor (CYF) models like those used in earlier studies, or more sophisticated crop growth models—must be customized to the diverse crops and cropping systems found in the BNH. Moreover, this baseline requires improved local information: regional estimates of climate and hydrological factors affecting yield should be downscaled to the resolution of agroecological zones (e.g., Figure 7), in order to inform stakeholder analysis of the relationship between cropping practices, precipitation variability, and yield stability under highly localized climate conditions. 

Looking to future conditions, earth system scientists can work with agriculture researchers and practitioners to provide information on evolving patterns of climate variability at the local scale. This is not a trivial exercise. Downscaled temperature projections—which will be important when predicting localized crop performance potential in the steeply dissected terrain of the BNH—should be implemented using methods that have been validated for current conditions. Such work will require research that makes use of available *in situ* data records, that improves the calibration of statistical downscaling methods through meteorological monitoring campaigns, and that implements innovative techniques, including satellite-based downscaling [52,53], for the BNH. Communication of uncertainty will also be critical: models that are sensitive to precipitation inputs should be presented as scenarios for risk evaluation, given GCM divergence in prediction of future precipitation patterns in the region. At the same time, crop functions that are sensitive to temperature or to transient soil moisture stress might be amenable to more skillful projection at local scale, provided that physiographically based downscaling methods can be adequately calibrated.

Beyond the technical aspects of climate and crop yield analysis, earth system scientists need to engage in interdisciplinary teams in order to evaluate the adaptive potential of existing resilience strategies that farmers currently practice in the BNH, to link climate projections effectively to economic analysis, and to determine the most relevant time scales of analysis. Projections to 2099, for example, are not particularly relevant to today’s on-farm cropping decisions, but risk evaluations over the coming 1–2 decades—including assessment of the rate of anticipated change—are of real interest to extension agencies and to farming communities. In some cases, localized seasonal forecasts and early warning systems (EWS) may be the most critical ESS contribution to agricultural resilience: in a climate of changing patterns of variability, advance information for risk evaluation in planting and harvest decisions can help to avert climate-related agricultural losses. Large uncertainties in the second half of the 21st century, then, are not the primary limitation for ESS contributions to agricultural resilience; uncertainties in downscaling of near-term projections and predictive skill on the seasonal time scale are of greater relevance.

In summary, ESS can make a contribution to projections of agricultural productivity in the BNH, but this work will require: (1) improved crop models, customized for BNH crops and cropping systems and calibrated against field data; (2) climate reanalyses downscaled to a resolution that captures the diverse agroecological zones of the BNH—a task that can be accomplished using satellite data and physiographically-based downscaling procedures in combination with in situ data, but that has yet to be demonstrated in the BNH; and (3) application of crop models and downscaling techniques to near-term climate projections and, potentially, seasonal forecasts, to provide localized yield forecasts and climate change impacts analysis. These are all feasible tasks that build on ESS techniques that have demonstrated success in other regions.

### 4.2. Erosion Potential

Experts participating in the *Workshop on Climate Resilience in the Blue Nile Highlands* identified adequate characterization of current and future erosion rates in the BNH as a significant research need. Published estimates of erosion rates in the Ethiopian Highlands range from 16–300 tons·ha^−1^·year^−1^ [37,39,55]. On a basin scale, these estimates can be constrained by measurements of sediment in the river, but the distribution of soil erosion across topography and land cover is poorly understood. Given this lack of information, combined with the known sensitivities of erosion rates to climate variability and geographic setting, the first task for ESS collaborations is to establish a more robust baseline of spatially distributed erosion rates across the BNH, including upland erosion, gulley wasting [38,56], and bluff erosion in the Blue Nile gorge. This work will build on background estimates available from earlier basin-wide assessments, promising emerging modeling approaches [57], and available field site analysis of erosivity and erosion rates [30,36,37]. A number of groups are actively working on soil conservation strategies within the Ethiopian Highlands; a detailed distributed map of potential and actual erosion rates will inform efforts to expand current work to high priority sites across the BNH. 

Key ESS tools in this evaluation include satellite-based landscape classification [58] and erosion monitoring [59], incorporation of demonstrated soil conservation strategies into probabilistic, physically-based land degradation models, sediment transport analysis [60], and controlled field investigations of erosion rates under a range of landscape settings and soil management practices. It is also important to understand how erosion affects local farmers, including through impacts on sustainable crop yields [40].

Precipitation intensity is a critical input to physically-based soil loss models, and it is a variable that is poorly constrained in present observations and future climate projections [28]. Climate scientists and hydrologists should engage in monitoring campaigns to provide better estimates of precipitation intensity across topography under current conditions, with the expectation that targeted *in situ* monitoring can be upscaled using satellite information [61,62]. The sensitivity of erosion estimates to observed precipitation extremes will provide a constraint on risk assessment for future erosion potential under a range of scenarios of precipitation intensity change. Scenarios of future erosion risk can then be approached both statistically, using cumulative distribution functions of observed precipitation intensities as a baseline [63], and dynamically, employing mesoscale atmospheric models to downscale GCM predictions. Dynamical downscaling will allow ESS researchers to investigate nonstationarities in localized climate extremes in an evolving climate system as well as to investigate the atmospheric dynamics that lead to intense convective events. But the uncertainty associated with such downscaled projections is extremely high. For both statistical and dynamical methods, cross-validation and data evaluation under current conditions is a high priority. When applied to climate projections, it is important that results of these downscaling analyses are clearly understood as tools of risk assessment rather than deterministic predictions of future conditions. 

In summary, the most pressing ESS research tasks to address erosion risk under climate change are studies of erosion processes today. First, collaborative efforts to generate and to ground truth satellite-informed land cover classifications are critical. These classifications should account for land use and cultivation practices, soil type, vegetation coverage, and slope characteristics [58], such that they can inform implementation of spatially-distributed soil loss models that have been calibrated at point scale within the BNH. Second, work on soil loss models—both for on-field erosion and gulley and bluff erosion—should be expanded to encompass the physiographic diversity of the BNH. Third, a network of high temporal frequency precipitation measurements should be established in order to provide a baseline of precipitation erosivity for the region. This network can then be employed to evaluate remote precipitation monitoring techniques—which, taken alone, tend to underestimate rainfall intensities in the Ethiopian highlands [60]—and to evaluate the simulation of precipitation processes in mesoscale atmospheric models typically applied to dynamical GCM downscaling. These baseline activities are required in order to provide a firm foundation for studies of future erosion risks.

### 4.3. Downstream Water Value

As noted in Section 3, the quantity and quality of flows from the BNH are of considerable interest to downstream stakeholders in Ethiopia, Sudan, and Egypt. The role of ESS in evaluating water quantity opportunities and risks associated with climate change and dam construction in the Blue Nile basin is well-established [35,51,64]. This line of research should continue in order to provide more refined estimates of the evolving water balance of the basin and associated variability in flow rates. Satellite-derived estimates of evapotranspiration [65,66] are being implemented for the region in order to evaluate current and potential future rates of water consumption across land cover and landscape classes. Hydrological model ensembles are also being applied in order to constrain spatially distributed estimates of runoff contributing to river flow [67]. From the perspective of BNH land management, such studies of the potential to assess the sensitivity of Nile River flows to changing watershed land use patterns in the headwaters region.

At the same time, improved estimates of sediment yield and erosion control opportunities, as described above, can provide an important contribution in the context of downstream water value. Scenario analysis of spatially distributed sediment delivery for a range of river development and watershed management strategies under climate change can provide objective information on the relationship between watershed development in the BNH and sediment load reaching infrastructure downstream in Ethiopia and Sudan. These analyses can be used to characterize risk to infrastructure as well as to identify high-value watershed management projects in the headwaters. Watershed management strategies that benefit communities of the BNH and downstream riparians are a significant opportunity area for transboundary cooperation and for motivating investment in climate resilient development.

The challenge of preserving and improving water value under climate change, then, is intimately related to resilience activities related to agricultural production and on-farm soil erosion. But as a transboundary basin issue, downstream water value analysis places a premium on integrated basin modeling, on trustworthy remote monitoring techniques, and on scenario studies that address the planning time horizon of large infrastructure development. Specific ESS research tasks include the development and calibration of basin-scale hydrological analysis systems that include credible estimates of sediment transport and agricultural productivity in addition to the standard basin model estimates of hydroelectric potential and irrigation demand. This is not a trivial task, given the aforementioned research needs in erosion and agriculture productivity. Nevertheless, researchers need to develop the framework for such integrated decision support, such that focused studies on erosion, agriculture, sediment transport, and climate variability can feed into an appropriate basin scale decision support system (DSS). The Nile Basin DSS (NBDSS) being developed by the Nile Basin Initiative is the preeminent effort to establish such a system (www.nilebasin.org). An open source version of the NBDSS will provide particular value, as it will leverage the contributions of the broader research community in support of collaborative water value analysis.

**Figure 8:**
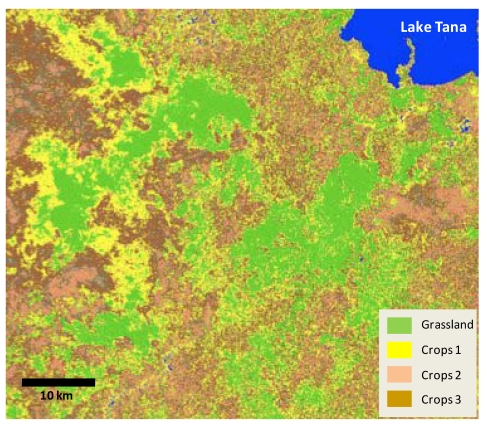
Satellite-based land cover classification for a region southwest of Lake Tana, produced at 30m resolution using images from the Landsat Thematic Mapper.

**Figure 9:**
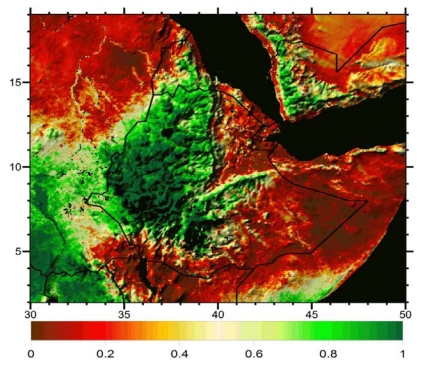
Ratio of actual evapotranspiration to potential evapotranspiration for the larger region that includes the BNH. Values are the June–September average for the year 2009, as derived using the ALEXI satellite-based algorithm [65,66].

For all three identified interaction points between local climate and land degradation—agricultural productivity, erosion, and downstream water value—an accurate baseline was identified as a key research need. *In situ* measurements are limited in spatial and temporal coverage, so merging data streams from physically-based models, satellite measurements, and available *in situ* stations is required. The principles of data merging and cross-comparison of independent techniques is currently being pursued under NASA’s *Project Nile* initiative, which is in the process of developing customized land cover maps (Figure 8) [58], water consumption estimates [66], and land data assimilation systems [54] that can be applied to hydrological analysis in the BNH. Innovative approaches to combining these independent sources of environmental information can be particularly valuable in data limited regions like the BNH. Satellite-derived evapotranspiration estimates, for example, can provide proxy validation for downscaled precipitation fields, while local contrasts in temporal evolution of the ratio of satellite-observed actual evapotranspiration to meteorological estimates of potential evaporation (e.g., Figure 9) may reflect contrasts in water holding capacity, which is sometimes a symptom of land degradation. When combined with satellite-derived land cover maps and topographic data, these estimates can be used to evaluate land degradation across agroecosystems and landforms. Through these customized analytical techniques, efforts like NASA’s Project Nile can provide a robust ESS baseline that serves as a platform for projecting future climate change impacts and evaluating resilience-building options.

### 4.4. Confronting Uncertainty in the BNH

For all of the promise that ESS holds for improved climate resilience strategies in the BNH, uncertainty in the climate forecast continues to present a daunting challenge. The problem of uncertain climate change impacts at regional scale, as noted earlier, is an active area of research for climate scientists and development experts [14]. For the BNH, experts participating in the climate resilience workshop identified a number of strategies for reducing and coping with uncertainty in adaptation planning.

The first point, as noted in sections 4.1 and 4.2, is that it is possible to distinguish between uncertainty in the climate forecast and uncertainties that stem from limitations in understanding or monitoring of present-day conditions. For certain applications—erosion risk, for example—the uncertainty associated with our limited understanding of present conditions is tremendous. ESS contributions to baseline analysis and to evaluation of intervention options are, for these applications, as important as or more important than studies of future change. A corollary to this point is the expectation that many ESS-informed resilience building interventions will be resilient to changes in climate over the coming decades—the efficacy of investments in cover crops, forest conservation, or drainage infrastructure, for example, are more sensitive to informed targeting and design and to effective engagement of local communities than they are to uncertainty in climate projections. 

A primary application of ESS, then, is to reduce present uncertainty through improved monitoring systems, field experiments that quantify water and energy fluxes and erosion potential, better characterization of the drivers of interannual climate variability, and adaptation of hydrological models to the conditions of the BNH (e.g., [68]) and crop models to the cultivation systems and crop types endemic to the region (e.g., [69,70]). A focus on improved monitoring, information systems, and models for the BNH will substantially reduce uncertainties relating to the sensitivity of natural and human systems to physical change. Satellite-based analysis has a clear role to play in this area. Cross-comparison of ALEXI water consumption and water use efficiency estimates with model-generated water balance estimates (Section 4.3) is one example of how satellite data can be used to improve water resource analysis and to inform model development. 

Of course, many adaptation activities are directly sensitive to uncertainty in GCM projections of future climate change. Decisions relating to crop breeding, biodiversity conservation, and construction of large infrastructure, for example, must consider predicted changes in temperature and precipitation. In approaching these adaptation challenges, it is important to focus on uncertainties in the variables most relevant to each decision process, evaluated over the time horizon of interest. The potential to make use of an uncertain forecast by focusing on variables of interest was addressed in Section 2.2. The case of large uncertainty in precipitation (Figure 2) versus reasonable certainty in temperature rise and increased soil moisture deficit (Figure 3) is a particularly relevant example for projecting crop conditions in the BNH. Time horizon also comes into play in the crop breeding example, since the range of climate projections increases significantly in the second half of the 21st century due to uncertainties in future fossil fuel emissions and in climate feedbacks. While a major hydroelectric project might need to be designed to account for a wide range of uncertainties on the 50–100 year time horizon, a crop breeding project may only need to consider relatively well-constrained predictions for the next few decades. 

Finally, the ensemble of GCM predictions available through CMIP archives does offer an opportunity for physically-based model downselection to narrow the range of uncertainty in climate projections. As noted in Section 2.1, model downselection is a challenging process that does not always yield improved projections [15,16,17,18]. Recent work, however, has suggested that East Africa is a region where model downselection is both necessary and feasible. This is the case because most GCMs included in the CMIP3 archive fail to reproduce observed climate variability and trends in East Africa over the past thirty years, and this failure can be attributed to the way in which models represent large scale atmospheric circulations affecting the region [71]. However, not all models suffer equally from this shortcoming. For example, in ongoing work we have identified the Geophysical Fluid Dynamics Laboratory Climate Model 2.1 (CM2.1) as an example of a GCM that has captured the recent drying trend in East Africa. CM2.1 is also known to be a model that represents ENSO, Indian Ocean warming, and Southern Ocean variability with reasonably good fidelity, such that CM2.1 21st century simulations might be expected to provide a reasonable projection of variability in large-scale processes affecting the BNH. Pending further evaluation, it may be possible to constrain CMIP5 projections of future climate variability and change in the BNH using such physically-based assessments of model performance. 

## 5. Conclusions

Climate resilient development is a major priority in the BNH, where vulnerability to climate variability and change is high and the regional implications of climate-induced damages could be severe [6,9]. But projections of future climate in the BNH are highly uncertain, limiting the utility of deterministic estimates of climate change impacts. In this context, ESS can contribute to climate resilience goals by establishing a robust baseline on the physical and biological impacts of climate variability, by informing adaptation options through scenario-based risk assessments, and by distinguishing between predictions of relatively high confidence (e.g., increased evaporative demand) and those of relatively low confidence (e.g., changes in annual precipitation).

Importantly, if the research is to have a lasting impact on climate adaptation, ESS studies of climate impacts and climate resilience options must be nested in the interdisciplinary, multi-stakeholder conversation on climate adaptation options. Risks and uncertainties identified in ESS projections are most meaningful when integrated with complementary studies of demographic change and economic and policy opportunities, informed by development priorities at the relevant scale of analysis. Work on such community-level resilience studies is presently underway in the BNH [6]. 

In this paper we have summarized and built upon ESS-related discussions at the recent *Workshop on Climate Change Resilience in the Blue Nile Highlands*. Land degradation—specifically, water-driven soil erosion—was identified as one of several high priority concerns for the region, and here we have identified a number of ways in which ESS analysis can inform efforts to halt and reverse land degradation in the context of climate change. When first approaching this issue, ESS participants focused on the most direct physical relationship between climate and erosion—the impact of local precipitation changes on mechanical soil mobilization and transport—while some participants from the social science and development communities began with the assumption that climate projections for the region are too uncertain to provide anything more than a justification for “no regrets” investments in development. Upon consideration, however, participants identified numerous points of intersection between ESS information and the coupled social/environmental processes related to land degradation. This framework points to a number of opportunities to apply ESS-informed baseline evaluation and risk assessment in support of informed adaptation strategies. Figure 6 is just one example of how the consultative exercise can produce a conceptual model that serves as a foundation for broader stakeholder discussion of a specific adaptation challenge.

Moving forward, the experiences shared at the *Workshop on Climate Resilience in the Blue Nile Highlands* are expected to catalyze broader and more active collaboration between earth system scientists and the broader climate resilience community. Through ongoing engagement, ESS information will be applied to inform climate resilience-building activities in the BNH. Experiences in this area will contribute to the growing understanding of resilience challenges and opportunities in regions facing an uncertain climate future.
